# *F. prausnitzii*-derived extracellular vesicles attenuate experimental colitis by regulating intestinal homeostasis in mice

**DOI:** 10.1186/s12934-023-02243-7

**Published:** 2023-11-15

**Authors:** Lin Ye, Yizhong Wang, Fangfei Xiao, Xufei Wang, Xiaolu Li, Rong Cao, Jiayue Zhang, Ting Zhang

**Affiliations:** 1 Department of Gastroenterology, Hepatology and Nutrition, Shanghai Children’s Hospital, Shanghai Jiao Tong University School of Medicine, 355 Luding Road, Shanghai, 200062 China; 2 Gut Microbiota and Metabolic Research Center, Institute of Pediatric Infection, Immunity and Critical Care Medicine, Shanghai Jiao Tong University School of Medicine, 355 Luding Road, Shanghai, 200062 China; 3 Shanghai Jiao Tong University School of Nursing, Shanghai, 200025 China

**Keywords:** *F. Prausnitzii*, Extracellular vesicles, Colitis, IBD

## Abstract

**Background:**

Emerging evidence has shown that extracellular vesicles (EVs) derived from gut bacteria play a crucial role in microbiota-host interactions. Here, we aimed to evaluate the attenuating effect of EVs derived from a reduced commensal bacterium, *F. prausnitzii* (*Fp*-EVs), in inflammatory bowel disease (IBD) on dextran sulfate sodium (DSS)-induced colitis in mice.

**Results:**

*Fp*-EVs isolated by ultracentrifugation and typically exhibited a double concave disc shape with an average diameter of 172 nm. *Fp*-EVs treatment reduced DSS-induced weight loss, disease activity index (DAI) score, colon length shortening, histological damage, neutrophil infiltration and increased intestinal epithelial apoptotic cells in DSS-induced colitis mice. Fp-EVs upregulated the protein expression of zona occludens (ZO)-1 and Occludin and increased the ratio of Tregs in the colon tissue of colitis mice. Furthermore, *Fp*-EVs downregulated the expression of the proinflammatory cytokines interleukin-1β (IL-1β), IL-2, IL-6, IL-12a, IL-17a, Interferon-γ (IFN-γ), tumor necrosis factor - α (TNF-α), granulocyte-macrophage colony stimulating factor (GM-CSF) and upregulated the anti-inflammatory cytokines IL-4, IL-10, and transforming growth factor β (TGF-β) in DSS-treated mice. Moreover, *Fp*-EV treatment markedly reduced the phosphorylation of these proteins Nuclear factor-κB (NF-κB) and Mitogen activated protein kinase (MAPK), and regulated the expression of nuclear factor erythroid 2-related factor (Nrf2) and heme oxygenase-1 (HO-1).

**Conclusion:**

Our findings revealed that *Fp*-EVs attenuated DSS-induced colitis by modulating the intestinal mucosal barrier function and immunological profile. Our findings reveal that *Fp*-EVs attenuate DSS-induced colitis by modulating intestinal mucosal barrier function and the immunological profile.

**Supplementary Information:**

The online version contains supplementary material available at 10.1186/s12934-023-02243-7.

## Introduction

Inflammatory bowel disease (IBD) is a multifactorial progressive disease characterized by chronic and recurrent inflammation of the gastrointestinal (GI) tract, disturbance of the gut microbiome and exacerbated immune responses [1, 2]. IBD affects people of all ages worldwide, the global prevalence of IBD has been increasing in recent years, with a higher prevalence in females than in males [3]. Although the precise aetiology of IBD is unclear, compelling evidence have indicated that multiple factors are involved in the pathogenesis of IBD, including genetic susceptibility, dysregulated immune responses, microbial dysbiosis and environmental factors [4].

The human gut harbors the most abundant and complicated microbial community, collectively referred to as the gut microbiota. Metagenomic studies have identified widespread gut microbiota disturbances and dysfunction in IBD patients [5], which is characterized by a deficiency of beneficial bacteria. *Faecalibacterium prausnitzii* (*F. prausnitzii*), the major bacterium of the *Clostridium leptum* group, is one of the predominant anaerobic bacteria in the human gut, accounting for 3–5% of the total gut bacteria [6]. Accumulating evidence has shown that the abundance of *F. prausnitzii* is significantly decreased in IBD patients compared to that in healthy controls and is related to the risk of disease recurrence [7, 8]. In addition, some recent studies have indicated that oral administration of *F. prausnitzii* could ameliorate the phenotype of experimental colitis mice as well as histopathological cryptitis and crypt abscesses [9]. Nevertheless, the mechanisms by which *F. prausnitzii* regulates the intestinal mucosal barrier and immune responses are not fully understood.

Extracellular vesicles (EVs) are heterogeneous, membranous natural nanoparticles released by all organisms, including bacteria, archaea, fungi, and parasites [10]. Multiple bacteria can constitutively generate EVs, which are spherical lipid bilayers with an average diameter in the range of 20 to 200 nm [11, 12]. The EVs generated from bacteria are used to acquire nutrients, defend against other microbes, resist the pressures of the host immune system, and facilitate adaptation to unique surroundings [13]. More importantly, EVs play a significant role in regulating intercellular communication by shuttling various molecules, including DNA, RNA, lipids, metabolites, and cytosolic and cell-surface proteins, from parental cells to other cells [14]. EVs can also directly interact with intestinal epithelial cells (IECs) and immune cells in the gut to modulate multiple signaling pathways involved in host‒pathogen interactions. Over the past two decades, EVs have emerged as a potential candidate for the treatment of refractory IBD because of their acellular nature, wide range of sources, and various biological characteristics. In this study, we aimed to evaluate the attenuating effect of EVs derived from *F. prausnitzii* (*Fp*-EVs) on DSS-induced colitis and further investigated the underlying mechanisms by examining intestinal barrier integrity and immunological profiles.

## Materials and methods

### ***F. prausnitzii*** culture and EV isolation

*F. prausnitzii* (ATCC 27,766) was purchased from the American Type Culture Collection (ATCC) and cultured anaerobically in LYHBHI medium (brain-heart infusion (37 g/L, Oxford, USA), yeast extract (5 g/L, Oxford, USA) and hemin (5 mg/L, Sigma)) supplemented with cellobiose (1 g/L, Oxford, USA), maltose (1 g/L, Oxford, USA), and cysteine (0.5 g/L, Sigma)) at 37 °C for 24–48 h [15–17]. The concentration of live bacteria in the exponential phase was calculated based on the optical density (OD) at 600 nm. Briefly, live bacteria were collected by centrifugation at 3000 rpm and 4 °C for 10 min. The bacterial culture supernatant was collected after centrifugation at 3000 rpm and 4 °C for 10 min and filtered to remove bacterial debris (0.45 μm) and contaminated proteins (0.22 μm). The filtrate was pelleted by ultracentrifugation in a 32 Ti rotor (Beckman Coulter, Fullerton, CA, USA) at 150,000 ×g for 2 h at 4 °C [18]. The pellets were resuspended in sterile phosphate-buffered saline (PBS), and the protein concentration of *Fp*-EVs was quantified using a BCA assay kit (Beyotime Biotechnology, China).

### Transmission electron microscopy and nanoparticle tracking analysis

**Fig. 1:**
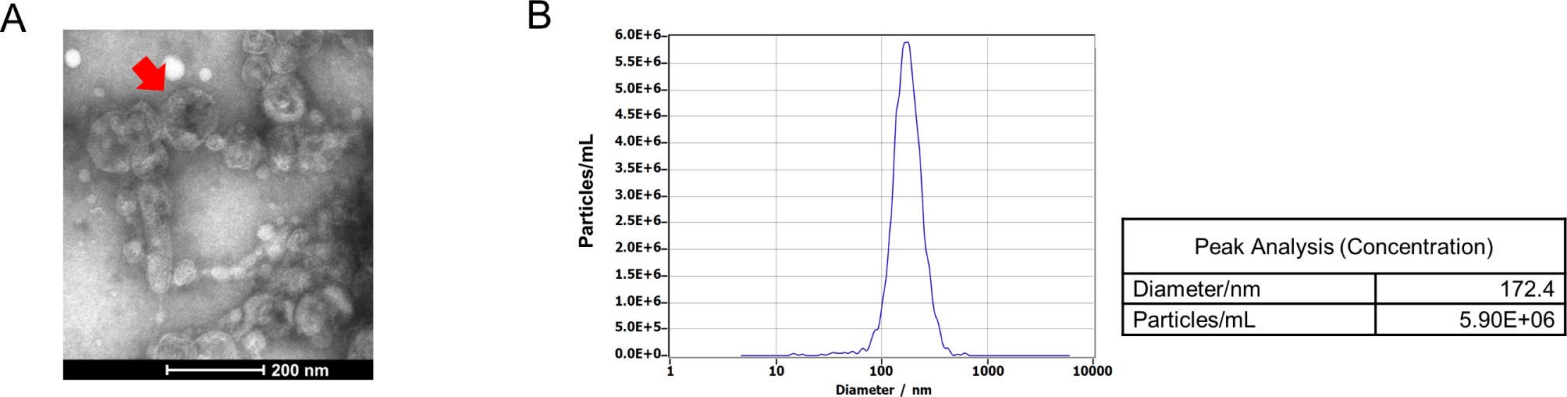
Identification and characterization of *Fp*-EVs. (**A**) TEM images of *Fp*-EVs. Scale bars represent 200 nm. (**B**) Size distribution analysis of *Fp*-EVs by NTA. TEM: transmission electron microscopy; NTA: nanoparticle tracking analysis

The morphology of EVs was visualized using transmission electron microscopy (TEM). Briefly, 10 μL of EVs was loaded onto a copper grid covered with formvar film and incubated for 1 min on ice. After negative staining with 2% phosphotungstic acid, the EVs were imaged using a HITACHI H-7650 transmission electron microscope. The size distribution was analyzed by nanoparticle tracking analysis (NTA) using ZetaView PMX 110 (Particle Metrix, Meerbusch, Germany) (Fig. 1).

### Experimental animals and Colitis induction

**Fig. 2:**
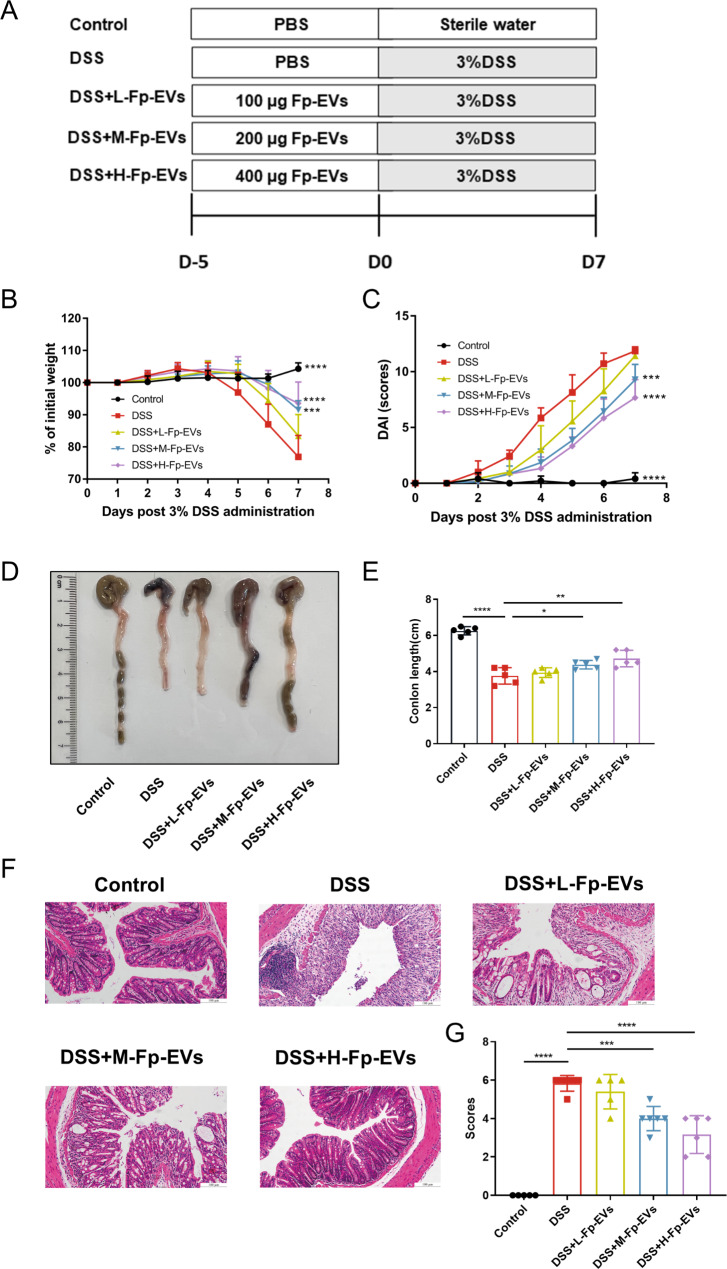
*Fp*-EVs attenuate DSS-induced colitis in mice. (**A**) Flow diagram illustrating the *Fp*-EV treatment schedule in DSS-induced mice (5–6 mice/group). (**B**) Changes in body weight among groups. (**C**) Changes in the disease activity index of the mice after the administration of 3% DSS. (**D**) Representative images of the colon. (**E**) Comparison of colon length among groups. (**F**) Representative H&E-stained colon sections. Magnification 200×. Scale bars represent 100 μm. (**G**) Histopathological scores among the different groups. Data are presented as the mean ± SD (n = 5–6). ** p < 0.01, ***p < 0.001 and ****p < 0.0001 vs. DSS group. DSS: dextran sulfate sodium; *Fp*: *Faecalibacterium prausnitzii*

All animal experiments were approved by the Experimental Animal Ethical Committee of Shanghai Children’s Hospital, affiliated with the School of Medicine, Shanghai Jiao Tong University (No. 2,018,008). Female C57BL/6 mice (6 weeks old, 18–20 g) were obtained from Ziyuan Lab (Zhejiang, China) and housed in ventilated cages under a 12:12 h light-dark cycle at a constant temperature (21–23 °C) with free access to food and water. After acclimatization for 1 week, the mice were randomly allocated into five subgroups: control group, dextran sulfate sodium (DSS) group, DSS + L-*Fp*-EV group (DSS + 100 μg of *Fp*-EVs, low dose), DSS + M-*Fp*-EV group (DSS + 200 μg of *Fp*-EVs, middle dose) and DSS + H- *Fp*-EV group (DSS + 400 μg of *Fp*-EVs, high dose). In the first 5 days, the mice in the DSS + *Fp*-EV groups were orally administered different doses of *Fp*-EVs once a day, while the other groups received the same volume of sterile PBS. For the next 7 days, all groups, except for the control group, were treated with 3.0% (w/v) DSS (molecular weight: 36–50 kDa, MP Biomedicals, Santa Ana, CA, USA) in their drinking water to induce colitis (Fig. 2A). Moreover, we also tested the therapeutic application of *Fp*-EVs through administered with *Fp*-EVs during or after DSS-induced colitis in mice (Figure S1A). Furthermore, wo also investigated the protective effect of *Fp*-EVs on 2,4,6-trinitrobenzene sulfonic acid (TNBS) - induced colitis mice. Female C57BL/6 mice were pretreated with *Fp*-EVs for 5 days, then the mice were administered 2.5% TNBS via rectal perfusion to imitate colitis, as previously described (Figure S2A) [19]. In addition, in order to assess the effect of LYHBHI itself on DSS-induced colitis in mice, two additional groups were added and were preadministered uncultured LYHBHI that underwent the same protocol as that for EV collection (ultracentrifugation): one with fresh drinking water and the other with drinking water containing DSS (Figure S3A). The disease activity index (DAI) was calculated based on the body weight loss, stool consistency, and the degree of intestinal bleeding as previously described [19]. At the end of the experiment, the mice were anaesthetized with 1% pentobarbital and sacrificed by cervical dislocation. Peripheral blood was obtained from the retro-orbital sinus, and serum was collected by centrifugation at 3,000 rpm for 15 min at room temperature and stored at ‒20 °C. The colon was extracted and the distance from cecum to anus was measured. The distal colon tissues were fixed in 4% paraformaldehyde for pathological, immunohistochemical and immunofluorescence examinations.

### Histopathological analysis of the colon

Paraffin-embedded colon tissue was sliced into 4–6 μm sections and subjected to haematoxylin and eosin (H&E) staining. The histopathological scoring of colon lesions was performed under a microscope by two pathologists in a double-blinded manner according to the scoring criteria. The sum of the two subscores resulted in a combined score ranging from 0 (no change) to 6 (widespread cellular infiltration and extensive tissue damage) [19]. The morphological images were captured using a digital microscope (Olympus BX43, Japan).

### Immunohistochemistry and immunofluorescence staining

After paraffin removal and dehydration, antigens were retrieved by boiling the tissue section in sodium citrate buffer, and endogenous peroxidase activity was blocked by incubation in 3% hydrogen peroxide solution. For immunohistochemistry, the colonic tissue sections were incubated with 5% BSA for 30 min at room temperature to block nonspecific binding, followed by staining with the primary antibody against myeloperoxidase (MPO) (Abcam, UK) overnight at 4 °C. After incubation with the secondary antibody (Abcam), the sections were stained with 3-diaminobenzidine and haematoxylin was used for nuclear counterstaining. The sections were visualized under a light microscope (Olympus BX43).

For immunofluorescence staining, sections were blocked with 5% BSA and incubated with primary antibodies against zonal occludens-1 (ZO-1) (Abcam, UK) and Occludin (Abcam, UK) overnight at 4 °C. The sections were washed with PBST and incubated with a biotinylated secondary antibody for 2 h at room temperature. A fluorescently labelled secondary antibody (Abcam) was used for fluorescent staining, and the nuclei were stained with 4′,6-diamidino-2-phenylindole (DAPI). The images were visualized and captured using a confocal microscope (Leica DMi8, Germany).

### Transferase-mediated deoxyuridine triphosphate-biotin nick end labeling (TUNEL) assay

Tunel assay was performed to detected the apoptosis cells in the colonic tisssue using an In Situ Cell Death Detection Kit (Roche, China) according to the manufacturer’s protocol. The images were visualized and captured using a confocal microscope (Leica DMi8, Germany).

### Quantitative real-time PCR

Total RNA was extracted from mouse colons using the SV Total RNA Isolation Kit (Promega Corporation, USA) following the manufacturer’s instructions. The concentration of RNA was quantified by a NanoDrop 2000 spectrophotometer (Thermo Fisher Scientific, USA). Total RNA (1000 ng) was converted into cDNA with the PrimeScript™ RT Master Mix reagent kit (Takara, Japan). The gene expression of interleukin-1β (IL-1β), IL-2, IL-4, IL-6, IL-10, IL-12a, IL-17a, interferon-γ (IFN-γ), tumour necrosis factor- α (TNF-α), granulocyte-macrophage colony stimulating factor (GM-CSF), transforming growth factor β (TGF-β) and β-actin was detected with SYBR Green Master Mix (Applied Biosystems, USA) using a Step One Plus TM Real-Time PCR Instrument (Applied Biosystems, USA). Primers used are listed in Supplementary Table 1. All procedures were performed in accordance with the manufacturer’s instructions. β-Actin was used as a housing gene for normalization, and data analysis was conducted using the 2 − ΔΔCt method.

### Western blotting analysis

For immunoblotting, total protein was extracted from colonic tissues using radioimmunoprecipitation assay (RIPA) lysis buffer containing protease and phosphatase inhibitor cocktail (Beyotime Biotechnology, China). The protein concentration was quantified using a BCA assay kit (Beyotime Biotechnology). Equal amounts of protein (30 μg) were subjected to sodium dodecyl sulfate–polyacrylamide gel electrophoresis (SDS–PAGE) and electrotransferred to a polyvinylidene difluoride (PVDF) membrane (Millipore, USA). The membrane was blocked with 5% nonfat milk for 2 h at room temerature and incubated overnight at 4 °C with the following primary antibodies: β-actin (Cell Signaling Technology, USA), zona occludens (ZO)-1 (Bioworld, USA), Occludin (Bioworld), nuclear factor erythroid 2-related factor (Nrf2) (ABclonal, USA), heme oxygenase-1 (HO-1) (ABclonal, USA), p-NFκB, NFκB, p-JNK, JNK, p-P38 and P38 (Cell Signaling Technology). Subsequently, the primary antibodies were detected with horseradish peroxidase-conjugated IgG secondary antibody, followed by a chemiluminescence system (Pierce, USA) and ChemiDoc XRS + imaging system (Bio-Rad, USA). Protein expression was normalized to that of β-actin in the same sample. The relative band intensities were quantified using Image Lab Version 2.0.1 (Bio-Rad).

### Enzyme-linked immunosorbent assays (ELISAs) for cytokine determination

The levels of serum cytokines IL-1β, IL-6, TNF-α and IL-10 were measured using commercially available ELISA kits (4 A Biotech Co. Ltd., China) following the manufacturer’s instructions.

### Flow cytometric analysis

The colon tissue collected from 0.5 cm below the caecum to 0.5 cm above the anus was cut longitudinally into 0.5 cm pieces after removing the remaining adipose tissue, mesenteric connective tissue, and Peyer’s patches. After washing with PBS, the colon pieces were digested with digest solution containing 1 mg/mL collagenase VIII and 1 U/mL DNase I (Gibco, USA) for 55 min in a 37 °C shaker. The supernatant was filtered through a 100 μm cell strainer and centrifuged at 2000 rpm for 5 min. The cell pellet was resuspended in RPMI 1640 containing 10% foetal bovine serum (FBS) after washing with PBS. Lamina propria lymphocytes (LPLs) were separated by density gradient centrifugation (cells were resuspended in 40% Percoll solution and overlaid with 80% Percoll solution at 2500 rpm for 25 min). The interface containing the LPLs was aspirated, washed with medium, and subjected to staining with anti-CD4 (eBioscience, USA) and anti-CD25 (eBioscience) at 4 °C in the dark for 30 min. After washing, the cells were fixed and permeabilized with 200 μL of fixation-permeabilization buffer overnight at 4 °C in the dark. Subsequently, the cells were incubated with Foxp3-PE (eBioscience) at 4 °C in the dark for 1 h and analyzed by flow cytometry (Cyto Flex S). The data were analyzed using FlowJo software (Tree Star).

### Proteomic analysis

The *Fp*-EVs were obtained and processed for label-free proteomic analysis by Applied Protein Technology (Shanghai, China). Briefly, total proteins of *Fp-*EVs were extracted and digested overnight with trypsin according to the filter-aided sample preparation (FASP) procedure described by Matthias Mann [20]. Liquid Chromatography-Mass Spectrometry/ Mass Spectrometry (LC‒MS/MS) analysis was performed on a Q Exactive mass spectrometer (Thermo Scientific) that was coupled to an Easy nLC (Thermo Fisher Scientific) for 60/120/240 min. The peptides were loaded onto a reversed-phase trap column (Thermo Scientific Acclaim PepMap100, 100 μm*2 cm, nanoViper C18) connected to a C18 reversed-phase analytical column (Thermo Scientific Easy Column, 10 cm long, 75 μm inner diameter, 3 μm resin) in buffer A (0.1% formic acid) and separated with a linear gradient of buffer B (84% acetonitrile and 0.1% formic acid) at a flow rate of 300 nl/min controlled by IntelliFlow technology. The MS raw data were combined and searched using the MaxQuant 1.5.3.17 software for identification and quantitative analysis. Tandem mass spectra were searched against the UniProt *F. prausnitzii* database concatenated with a reverse decoy database. The false discovery rates at the protein and peptide-to-spectrum match levels were adjusted to < 1%. Gene Ontology (GO) analysis was conducted to annotate all the identified proteins. Functional enrichment analyses based on GO and Kyoto Encyclopedia of Genes and Genomes (KEGG) pathways were performed to better understand the functions of the proteins in the *Fp-EVs*.

### Statistical analysis

The data are expressed as the mean ± standard deviation (SD). One-way analysis of variance (ANOVA) followed by Tukey’s test was performed to assess the significance of differences among the groups. Statistical significance was set at P < 0.05. Prism version 9.0 (GraphPad Software Inc., USA) was used for the statistical analysis.

## Results

### Characterization and proteomic analysis of ***Fp***-EVs

*Fp*-EVs were obtained by ultracentrifugation and characterized using TEM and NTA. As shown in Fig. 1A and B, electron microscopy observations of the isolated *Fp*-EVs showed a typical double concave disc shape with a size distribution that ranged from 10 to 200 nm with an average diameter of 172 nm.

### ***Fp***-EVs attenuate experimental colitis in mice

To examine the protective effect of *Fp*-EVs on DSS-induced colitis in mice, we treated mice with *Fp*-EVs for 5 days prior to DSS administration (Fig. 2A). As shown in Fig. 2, *Fp*-EV pretreatment markedly prevented body weight loss (Fig. 2B), bloody diarrhoea and decreased the DAI scores (Fig. 2C) and colon length shortening (Fig. 2D and E) in DSS-induced colitis mice in a dose-dependent manner. *Fp*-EVs partially attenuated intestinal mucosal damage and reduced inflammatory cell infiltration. Consistently, histopathology scores were significantly decreased in DSS-induced colitis mice treated with *Fp*-EVs (Fig. 2F and G). The therapeutic use of *Fp*-EVs can also mitigate DSS-induced colitis in mice (Figures S1). Similarily, pretreatment with *Fp*-EVs also significantly alleviated colonic injury and improved the survival rate of TNBS-induced colitis in mice (Figures S2). In addition, LYHBHI medium itself had no inhibitory effect on DSS-induced colitis (Figures S3).

### ***Fp***-EVs enhance intestinal mucosal barrier function

**Fig. 3:**
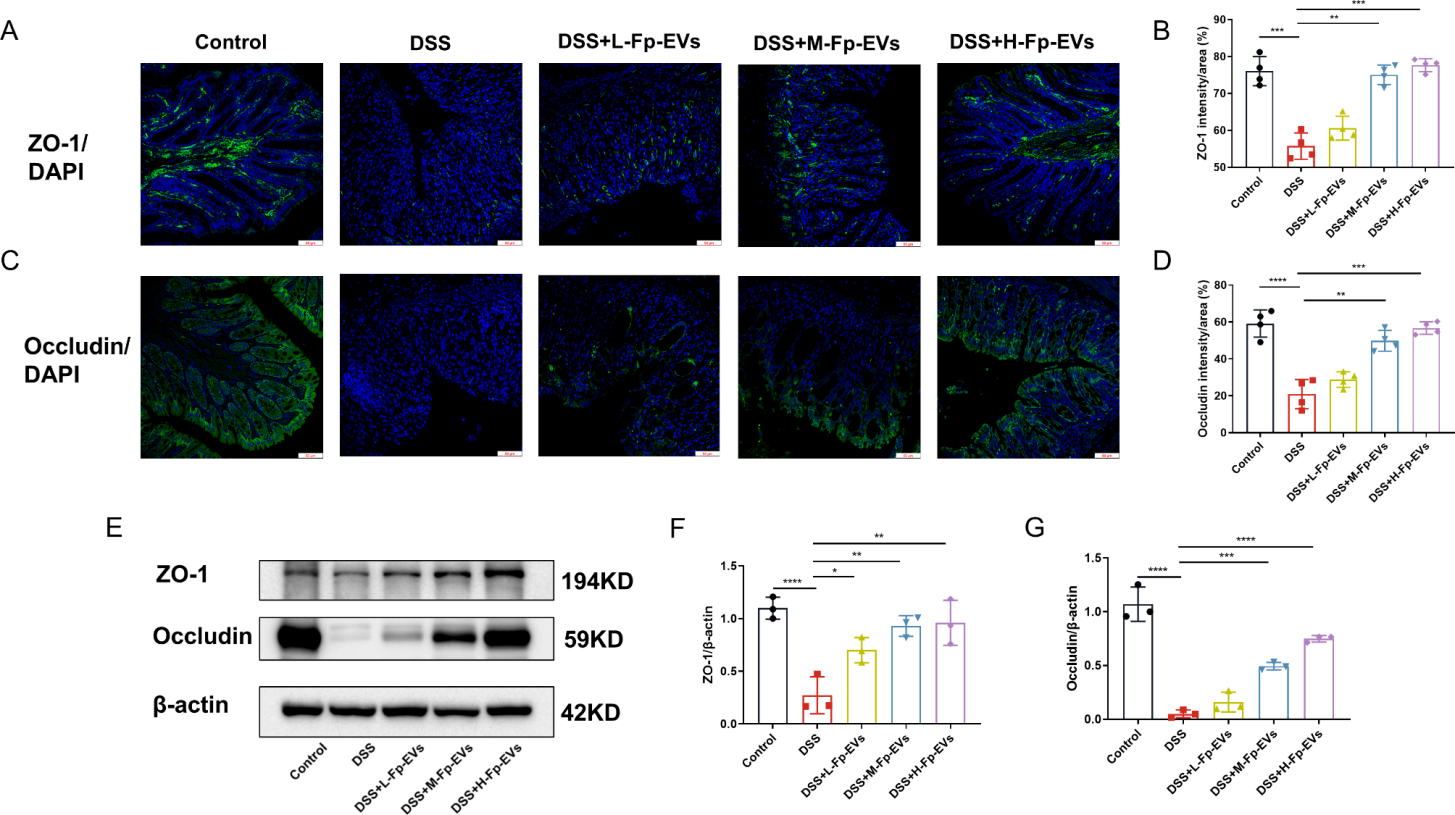
*Fp*-EVs maintain intestinal mucosal barrier function in DSS-induced colitis mice (3–4 mice/group). (**A**) and (**C**) Immunofluorescent staining of ZO-1 and Occludin in the colon. Magnification 400×. Scale bars represent 50 μm. (**B**) and (**D**) Analysis of the intensity/area of ZO-1 and Occludin among the different groups. (**E**) Protein levels of Occludin in colon tissue analyzed by western blotting normalized to β-actin. (**F**) and (**G**) Quantification of ZO-1 and Occludin calculated by the densities of the protein bands using Image Lab. Data are presented as the mean ± SD (n = 3–4). *p < 0.05, ** p < 0.01, ***p < 0.001 and ****p < 0.0001 vs. DSS group

The intestinal mucosal barrier is mainly composed of tight junction (TJ) proteins, which mediate robust mechanical stability, participate in various intracellular signaling pathways and help preserve tissue homeostasis [21]. TJ proteins include peripheral membrane proteins and transmembrane proteins (such as ZO-1 and Occludin) [22]. To assess the effect of *Fp*-EVs on intestinal mucosal integrity, we histologically analyzed the expression of ZO-1 and Occludin using immune-fluorescence staining. As shown in Fig. 3, compact microvilli and a solid connection structure were observed in the control group. In contrast, in the DSS group, the microvilli became sparse and disordered and the epithelial TJs became blurred and loose. However, pretreatment with Fp-EVs ameliorated this damage in a dose-dependent manner, with repaired microvilli and rebuilt TJ connections (Fig. 3A-D). Furthermore, we examined ZO-1 and Occludin expression in colon tissue using western blotting. As presented in Fig. 3E, DSS markedly decreased the expression of ZO-1 and Occludin in the colon tissue compared to the control group, while *Fp*-EVs increased ZO-1 and Occludin protein levels in a dose-dependent manner (Fig. 3F and G).

### ***Fp***-EVs alleviate acute injury and the apoptosis of epithelial cells in the colon

**Fig. 4:**
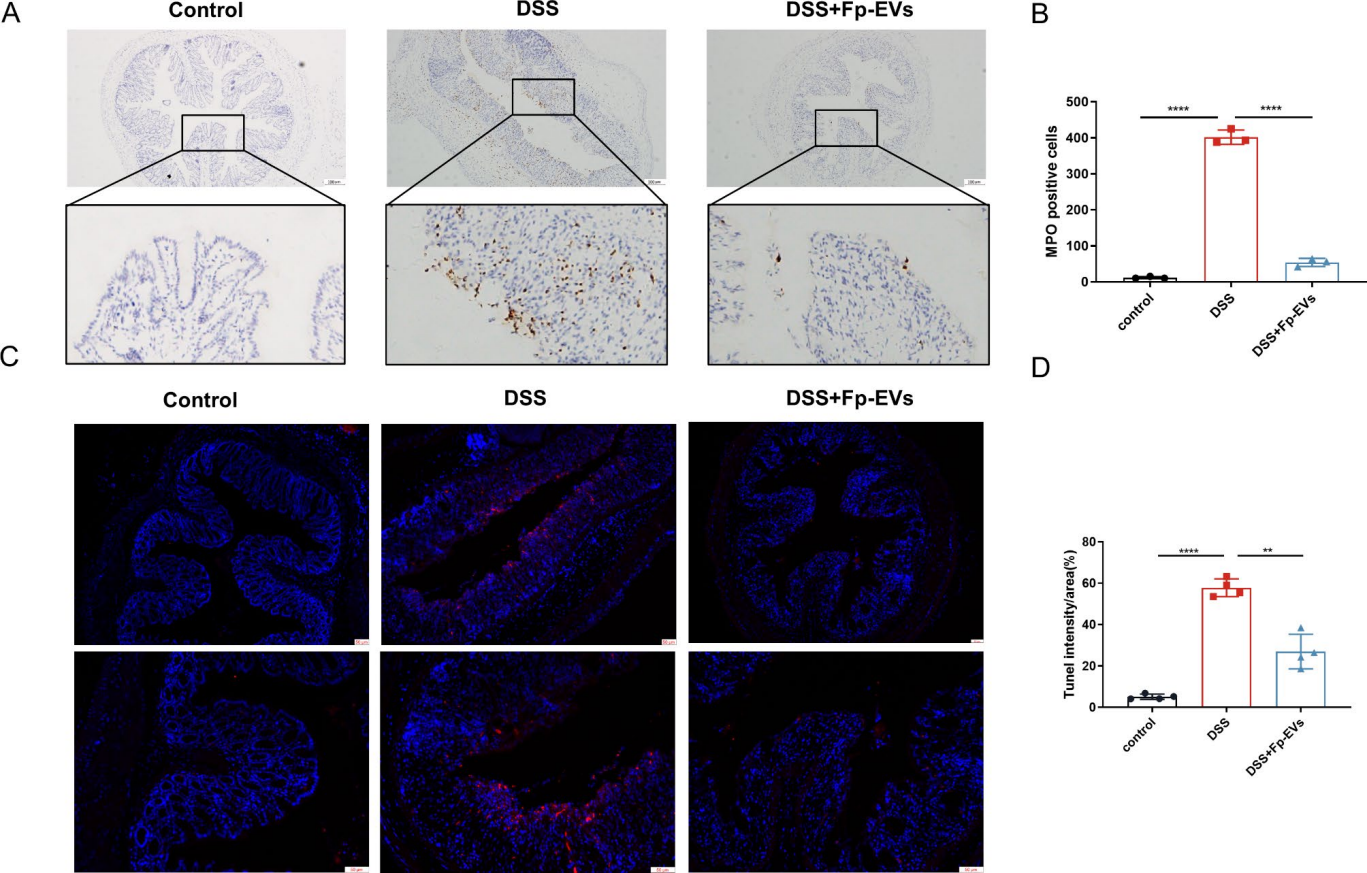
*Fp*-EVs alleviate acute colon injury and apoptosis of epithelial cells in the colon in DSS-induced colitis mice (3–4 mice/group). (**A**) Representative images of colon sections for MPO immunohistochemical analysis. Magnification 200×. Scale bars represent 100 μm. (**B**) Quantification of the number of MPO-positive cells among the different groups. (**C**) Tunel assay. Magnification 100×, 200×. Scale bars represent 50 μm. (**D**) Quantification of Tunel + cells (%). Data are presented as the mean ± SD (n = 3–4). *p < 0.05, ** p < 0.01, ***p < 0.001 and ****p < 0.0001 vs. DSS group. MPO: myeloperoxidase; TUNEL: Transferase-mediated deoxyuridine triphosphate-biotin nick end labelling

The enzyme myeloperoxidase (MPO) is present in neutrophils and is a crucial marker of acute inflammation and injury in the colon [23]. The semiquantitative immunohistochemistry data suggested that MPO expression was significantly elevated in mice treated with DSS compared to that in control mice, while treatment with *Fp*-EVs suppressed the elevation of MPO activity in DSS-induced colitis mice (Fig. 4A and B). In addition, the TUNEL assay further demonstrated that *Fp*-EVs treatment effectively repressed intestinal epithelial cell apoptosis induced by DSS (Fig. 4C and D).

### ***Fp***-EVs increase the ratio of CD4^+^CD25^+^Foxp3^+^ Tregs in colonic LPLs

**Fig. 5:**
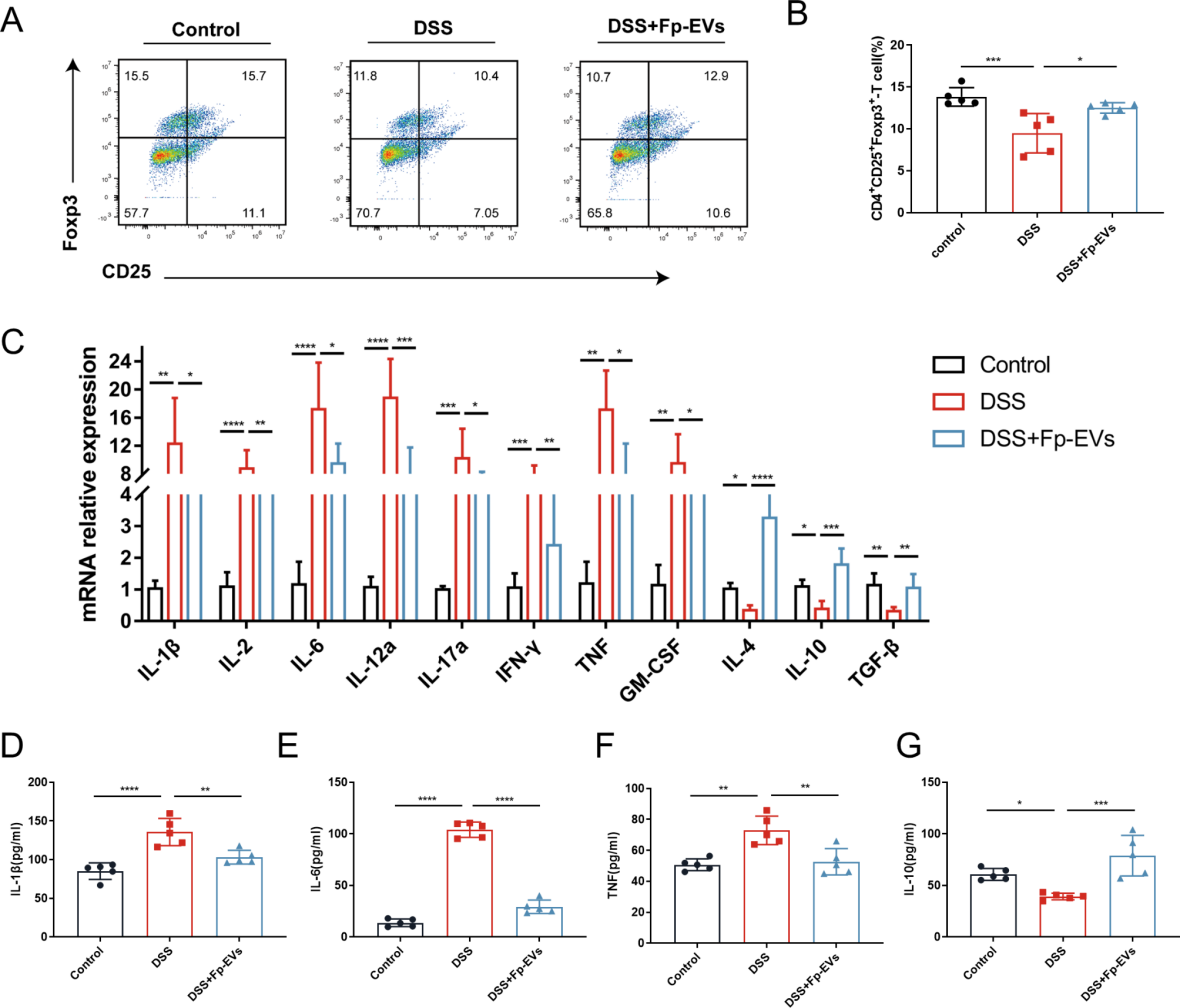
*Fp*-EVs modulate Tregs and inflammatory cytokine expression in DSS-induced colitis mice (5–6 mice/group). (**A**) Flow cytometry analysis of the ratio of CD4^+^CD25^+^Foxp3^+^ Tregs in colonic LPLs. (**B**) Bar charts showing the percentages of Tregs in colon samples from the control, DSS and DSS + *Fp*-EV groups. (**C**) mRNA expression of IL-1β, IL-2, IL-6, IL-12a, IL-17a, IFN-γ, TNF-α, GM-CSF, IL-4, IL-10 and TGF-β in colon tissue (normalized to β-actin). (**D**-**G**), Serum IL-6, IL-1β, TNF-α and IL-10 levels were measured by ELISA from the mice from the control, DSS and DSS + Fp-EV groups. Data are presented as the mean ± SD (n = 5–6). *p < 0.05 and ***p < 0.001 vs. DSS group. LPL: lamina propria lymphocyte

Regulatory T cells (Tregs) are crucially involved in the maintenance of gut mucosal homeostasis by suppressing abnormal immune responses against the commensal bacteria or dietary antigens. Thus, we examined the ratio of CD4^+^CD25^+^FOXP3^+^ Tregs in colonic LPLs from three groups of mice using flow cytometry. As shown in Fig. 5A and B, the proportion of CD4^+^CD25^+^FOXP3^+^ Tregs was significantly lower in the DSS group than in the control group, whereas *Fp*-EV treatment increased the ratio of CD4^+^CD25^+^FOXP3^+^ Tregs in the colonic tissue of DSS-treated mice.

### ***Fp***-EVs modulate cytokine expression

Cytokines play a pivotal role in the pathogenesis of IBD, as they regulate various inflammatory responses. In particular, the disequilibrium between proinflammatory and anti-inflammatory cytokines that occurs in IBD not only hinders the resolution of inflammation but also leads to tissue destruction and disease perpetuation [24]. The inflammatory molecules in the colon tissues were analyzed by real-time PCR. As shown in Fig. 5C, significant increases in the levels of various proinflammatory cytokines (IL-1β, IL-2, IL-6, IL-12a, IL-17a, IFN-γ, TNF-α and GM-CSF) and decreased levels of anti-inflammatory cytokines (IL-4, IL-10 and TGF-β) were observed in the colonic tissue of DSS-treated mice as compared with control group, while *Fp*-EV treatment could downregulate the expression of these proinflammatory cytokines and improved anti-inflammatory cytokine expression. The levels of IL-1β, IL-6, TNF-α and IL-10 in the serum of mice were measured using ELISA kits. As shown in Fig. 5D-G, there was elevated secretion of IL-1β, IL-6, and TNF-Α and reduced secretion of IL-10 in the serum of mice receiving DSS. However, pretreatment with *Fp*-EVs suppressed the levels of proinflammatory cytokines and promoted the expression of anti-inflammatory cytokines.

### ***Fp***-EVs suppresse the activation of NF-κB and MAPK and regulate the Nrf2/HO-1 signalling in DSS-induced colitis

**Fig. 6:**
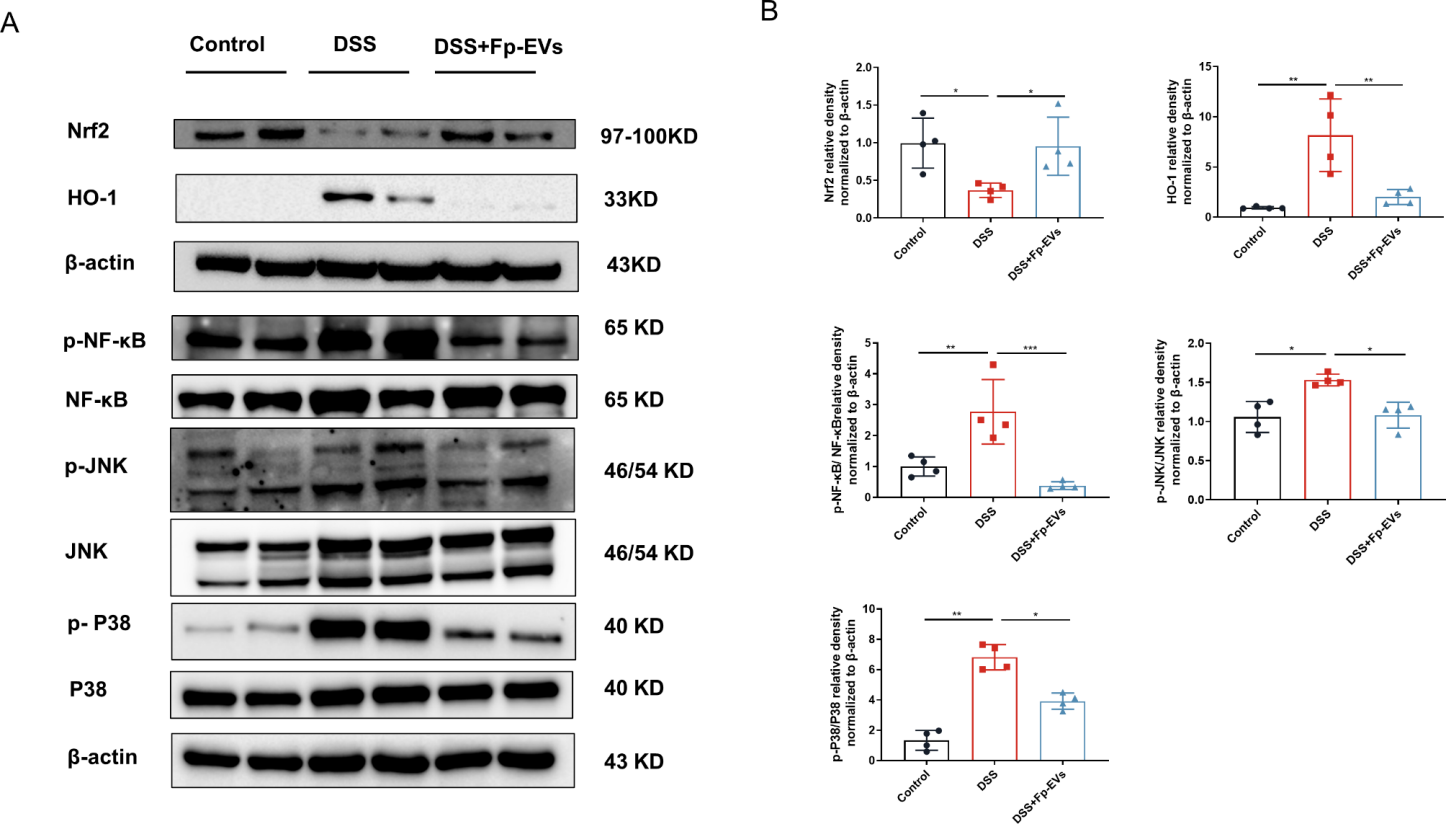
*Fp*-EVs suppresse the activation of NF-κB and MAPK and regulate the Nrf2/HO-1 signalling in DSS-induced colitis (3–4 mice/group). (**A**) Western blotting analysis of Nrf2, HO-1, p-NF-κB, NF-κB, p-JNK, JNK, p-P38 and P38 among groups. (**B**) Quantification of p-NF-Κb/NF-κB, p-JNK/JNK and p-P38 /P38 normalized to β-actin by calculating the densities of protein. Data are presented as the mean ± SD (n = 3–4). *p < 0.05, ** p < 0.01, ***p < 0.001 and ****p < 0.0001 vs. DSS group. Nrf2: nuclear factor erythroid 2-related factor, HO-1: heme oxygenase-1

Nuclear factor-κB (NF-κB) is an important nuclear transcription factor and the nuclear translocation of NF-κB promotes the production of pro-inflammatory factors TNF-α, IL-1β and IL-6. Phosphorylation / activation of NF-κB (p-NF-κB) leads to the nuclear translocation of NF-κB. Mitogen activated protein kinase (MAPK), a series of highly conserved serine / threonine protein kinases, also plays an important role in the pathogenesis of IBD. Thus, we detected the activities of p-NF-κB, NF-κB, p-JNK, JNK, p-P38 and P38 in these groups. Western blotting analysis showed that DSS induced the upregulation of p-NFκB, p-JNK and p-P38 as compared with the control group (Fig. 6A), while *Fp*-EV treatment could markedly reduce the phosphorylation levels of these proteins (Fig. 6B).

The occurrence of IBD is usually accompanied by an increase in reactive oxygen species (ROS), which causes irreversible damage to tissues. Nrf2 and its downstream target gene, HO-1, are often thought to play an important role in antioxidant stress. In our study, we found that Nrf2 was downregulated and HO-1 was upregulated in the colons of the mice in the DSS group, while *Fp*-EV treatment reversed the expression of Nrf2 and HO-1, thus playing the role of antioxidant stress (Fig. 6).

### Proteomic analysis of ***Fp***-EVs

**Fig. 7:**
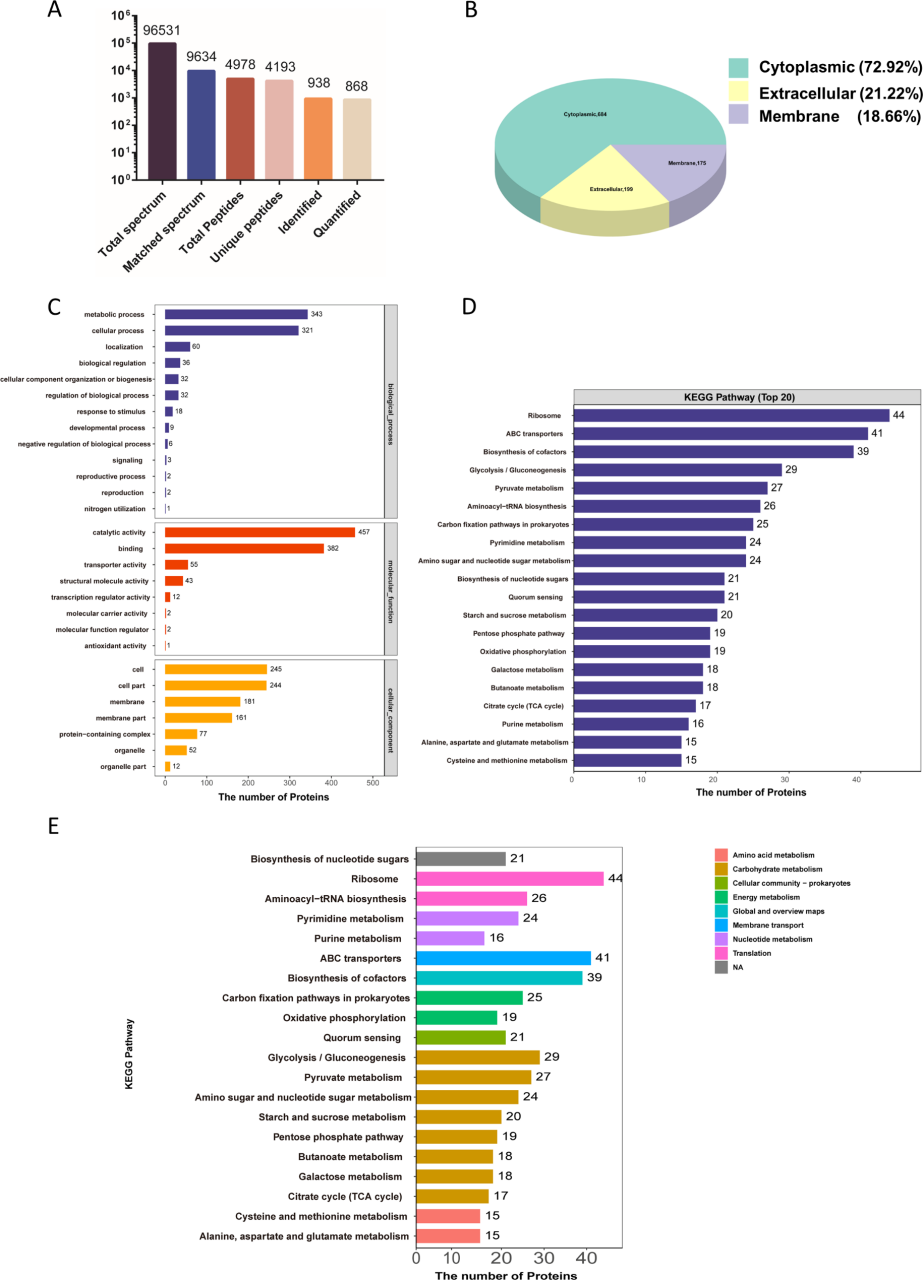
Proteomic analysis of *Fp*-EVs. (**A**) Summary of the tandem mass spectrometry database search results for *Fp*-EVs. (**B**) GO annotation of the proteins in *Fp*-EVs in terms of their subcellular localization. (**C**) GO biological process enrichment analysis of the proteins in *Fp*-EVs. (**D**) Top 20 enriched KEGG pathways. (**E**) Biological function pathway enrichment results at different levels. GO: Gene Ontology; KEGG: Kyoto Encyclopedia of Genes and Genomes

To further investigate the effect of *Fp*-EVs protein components on IBD, we conducted label-free proteomic analysis of *Fp*-EVs. A total of 938 proteins were identified in *Fp*-EVs by using label-free proteomic analysis, among which 868 proteins were quantified (Fig. 7A). Gene Ontology (GO) annotation was conducted to classify the proteins highly enriched in *Fp*-EVs according to their subcellular localization. As shown in Figs. 7B and 684 proteins were annotated as cytoplasmic proteins, 199 were classified as extracellular, and 175 were identified as membrane proteins. In addition, some proteins had the possibility of multiple localizations. Furthermore, GO enrichment analysis shown that *Fp*-EVs were enriched with proteins involved in metabolic and cellular process, catalytic and transporter activity, binding and membrane (Fig. 7C). KEGG pathway enrichment analysis revealed that the proteins in the *Fp*-EVs were involved in ribosome, ABC transporters, biosynthesis of cofactors, various biosynthetic and metabolic processes and oxidative phosphorylation (Fig. 7D and E). Moreover, we found that *Fp*-EVs proteins were involved in many inflammatory signaling pathways, including the MAPK signaling pathway, HIF signaling pathway, PI3K/Akt signaling pathway and AMPK signaling pathway (Supplementary Table 2).

## Discussion

Alterations in the gut microbiota are not only the cause but also an important clinical manifestation of IBD [25]. In general, IBD patients tend to have reduced bacterial diversity and a disorganized gut microbiota structure compared with healthy controls. In healthy human feces, *Firmicutes* and *Bacteroidetes* were the dominant bacterial strains at the phylum level [26]. The abundance of a major member of *Firmicutes*, *F. prausnitzii*, is significantly reduced in IBD patients and is associated with the risk of disease recurrence [7, 8, 17]. Therefore, the role of *F. prausnitzii* in the development of IBD has attracted great attention. A large number of studies have shown that *F. prausnitzii* could provide energy to the colonocytes to maintain intestinal health, playing roles as an anti-inflammatory and barrier protector in IBD [17, 27–29]. Some recent studies also indicated that oral administration of *F. prausnitzii* could ameliorate the weight loss and disease activity in experimental colitis mice as well as histopathological cryptitis and crypt abscesses [9]. Further studies have suggested that not only the *F. prausnitzii* itself but also the culture supernatant of *F. prausnitzii* exhibits a significant effect in alleviating experimental colitis in mice [15, 30, 31]. Furthermore, *F. prausnitzii* strain A2-165 or its supernatant has been confirmed to possess anti-inflammatory properties by downregulating MPO and proinflammatory cytokines, regulating the differentiation and proliferation of helper T-cells, and protecting the intestinal epithelial barrier in colitis mouse models [31, 32]. In addition, some studies have shown that *F. prausnitzii* produced short-chain fatty acids (SCFAs), such as butyrate, to maintain the Th17/Treg balance and ameliorate colorectal colitis [30]. Moreover, a 15 kDa protein with anti-inflammatory properties produced by *F. prausnitzii* was found to inhibit the NF-κB pathway in intestinal epithelial cells to alleviate dinitrobenzene sulfonic acid (DNBS)-induced colitis in mice and restore the gut barrier and ZO-1 expression under diabetic conditions [33, 34]. However, the mechanisms by which *F. prausnitzii* regulates the intestinal mucosal barrier and immune responses are not fully understood.

EVs, with lipid bilayers, have received increasing attention as mediators of intercellular communication. The shedding of EVs is also ubiquitous in a diverse set of organisms as part of their normal physiology or acquired abnormalities [35]. Most of bacterial membrane vesicles have diverse functions, including virulence factor and eukaryotic host defence factor transport, DNA transfer, bacteriophage interception, cell detoxification and bacterial communication [36–39]. EVs released from bacteria can transport bacterial components to host cells over long distances to induce immune and metabolic responses by mediating the interactions between bacteria and host cells [40]. Owing to the complexity of the gut microbiota, the functional roles of EVs in modulating the intestinal state are different. For example, EVs from *Fusobacterium nucleatum* compromise the intestinal barrier by targeting the RIPK1-mediated epithelial cell death pathway [41]. Moreover, it was reported that the oral administration of EVs from the probiotic *Akkermansia muciniphila* could ameliorate DSS-induced IBD phenotypes, such as body weight loss, colon length shortening, and inflammatory cell infiltration in the colon wall, and reduce the production of proinflammatory factors (such as IL-6) [42]. Previous studies have shown that *Fp*-EVs have greater efficacy in decreasing the levels of inflammatory cytokines and increasing the levels of anti-inflammatory cytokines compared to *F. prausnitzii* in vitro [43]. Furthermore, another study demonstrated that *Fp*-EVs increased intestinal barrier permeability in Caco-2 cells [44]. However, the direct effects of EVs produced by *F. prausnitzii* on cellular physiology and intestinal barrier regulation remain unclear. Here, we found that EVs derived from *F. prausnitzii* could alleviate the DSS-induced colitis in mice as evidenced by reductions in DSS-induced weight loss, DAI score, colon length shortening, histological damage and neutrophil infiltration.

Epithelial cells that bind to intercellular junction proteins are critical to maintain the integrity of intestinal barrier [45]. As a pivotal component of the epithelial barrier, TJs could aid in maintaining intestinal homeostasis and protect the intestinal epithelial mucosa from constant threats from proinflammatory mediators, pathogenic bacteria and viruses. Accumulating evidence has revealed that the loss of barrier proteins is the initiating factor and could lead to increased levels of proinflammatory cytokines and immune dysregulation in IBD [46]. Impairment in the intestinal mucosal barrier function may exacerbate disease progression, and these tight junction proteins have become lucrative targets to alleviate IBD. Our research showed that *Fp*-EVs could increase the expression of ZO-1 and Occludin in the colons of DSS-induced colitis mice to some extent. Therefore, the restoration of intestinal barrier function may partially explain the protective effects of *Fp*-EVs on DSS-induced colitis in mice.

Host innate and adaptive immunity cooperate and compensate for each other to maintain equilibrium in the highly sophisticated gut ecosystem in a stringent and conserved manner [47]. Neutrophils, a component of the innate immune system, are thought to play a critical role in the maintenance of intestinal homeostasis [48]. Under pathological conditions, such as IBD, the excessive accumulation of activated neutrophils in the gut is associated with mucosal injury and debilitating disease symptoms. Evidence has suggested that overactivated neutrophils can further damage the colon by releasing reactive oxygen species (ROS) and MPO, accelerating the apoptosis of intestinal epithelial cells [49, 50]. In our study, we found that DSS administration markedly increased the number of MPO-positive cells and TUNEL- positive cells, while *Fp*-EVs pretreatment reduced the acute inflammatory damage and apoptosis.

T cells are key players in the adaptive immune response, including mainly effector T cells and regulatory T cells. Tregs make a great contribution in the effective treatment of colitis. Previous studies have revealed that transferring naive CD4^+^ T cells into Treg-deficient mice led to colitis, while Treg transfer into colitis rodents significantly relieved intestinal inflammation [51]. In our study, we observed that *Fp*-EVs could increase the ratio of CD4^+^CD25^+^FOXP3^+^ Tregs in the colon of DSS-induced colitis mice. These findings expand our current knowledge that EVs from the supernatant of *F. prausnitzii* could play an important role in regulating T-cell differentiation. Furthermore, an increasing number of studies have suggested that abnormal cytokine secretion is associated with IBD, either as a cause or a consequence of their plasma or local inflammation of the intestinal mucosa [52]. Our data showed that the upregulation of proinflammatory cytokines (IL-1β, IL-2, IL-6, IL-12a, IL-17a, IFN-γ, TNF-α and GM-CSF) in the serum and colon tissues of DSS-treated mice were reduced by *Fp*-EV administration, while *Fp*-EVs could enhance the downregulated expression of anti-inflammatory cytokines (IL-4, IL-10 and TGF-β), which further verified the protective effect of *Fp*-EVs on colitis in mice.

In general, the common features and main mediators of IBD occurrence and development are no more than oxidative stress and its persistent inflammation. Compelling evidence have shown that elevated peroxidation products and cytotoxic molecules in the intestinal mucosa of IBD patients can cause tissue damage [53]. Therefore, antioxidant and anti-inflammatory pathways are effective ways to treat IBD. Nrf2 is a cytoprotective nuclear transcription factor that plays a central role in the basal activities and coordinated sensing of target gene products, including antioxidant enzymes and the major detoxifying enzyme, which plays an important role in the modulation of oxidative stress [54]. Under basal conditions, cytosolic thiol antioxidants maintain the stability of inactive Nrf2 heterodimers. When cytosolic thiol is depleted by ROS, the heterodimer dissociates and Nrf2 translocated to the nucleus, where it drives the transcription of genes such as HO-1 [55]. Therefore, in response to oxidative stress damage induced by DSS, nuclear Nrf2 levels increase, while cytoplasmic Nrf2 levels decrease. In our study, the expression of Nrf2 was decreased and the expression of HO-1 was increased in DSS mice, whereas *Fp*-EVs treatment could reversed the expression of Nrf2 and HO-1. In addition, the occurrence and development of IBD is often accompanied by the activation of NF-κB and MAPK signaling pathways [56]. It is worth noting that Nrf2 has a very close relationship with NF-κB, and it has been reported that the important role of Nrf2 in reducing inflammatory response is closely related to its ability to antagonize NF-κB [57]. *Fp*-EVs treatment could significantly inhibit the phosphorylation of NFκB, JNK and P38 MAPK. Therefore, our findings demonstrated that *Fp*-EVs could protect against DSS-induced colitis by suppressing the activation of NF-κB and MAPK and regulating Nrf2/HO-1 signaling pathways.

According to the *Fp*-EVs proteomic analysis, the underlying molecular mechanism by which *Fp*-EVs protect against DSS-induced colitis in mice may be the transfer of some bio-active components of parental bacteria to intestinal epithelial cells or immune cells. GO enrichment analysis shown that *Fp*-EVs were enriched with proteins involved in metabolic and cellular process, catalytic and transporter activity, binding and membrane, supporting the notion that EVs act as important cellular material transporters between cells [58]. KEGG pathway enrichment analysis revealed that the proteins in the *Fp*-EVs were involved in ABC transporters, biosynthesis of cofactors, various biosynthetic and metabolic processes and oxidative phosphorylation. These findings indicate that *Fp*-EVs are selectively enriched with various functional proteins. Moreover, *Fp*-EVs contained proteins involved in many signal transduction pathways, including the MAPK signaling pathway, PI3K/Akt signaling pathway, AMPK signaling pathway and HIF signaling pathway, which mediate the activation of central signaling cascades involved in the inflammatory response and oxidative stress [59]. However, the exact mechanisms and which *Fp*-EVs component that contribute to the relief of colitis require further investigation.

## Conclusion

In summary, *Fp*-EV administration attenuated DSS-induced colitis in mice by modulating the intestinal mucosal barrier and immunological profiles. Further studies are needed to determine the roles of the specific *Fp*-EV components that mediate the intestinal anti-inflammatory effect.

### Electronic supplementary material

Below is the link to the electronic supplementary material.

**Supplementary Material 1: Supplementary Fig. 1**. *Fp*-EVs treat DSS-induced colitis in mice. (**A**) Flow diagram illustrating the *Fp*-EV treatment schedule in DSS-induced mice (5–6 mice/group). (**B**) Changes in body weight among groups. (**C**) Changes in the disease activity index of the mice after the administration of 3% DSS. (**D**) Representative images of the colon. (**E**) Comparison of colon length among groups. (**F**) The level of FITC-Dextran among groups. Data are presented as the mean ± SD (n = 5–6). ** p < 0.01, ***p < 0.001 and ****p < 0.0001 vs. DSS group. DSS: dextran sulfate sodium; *Fp*: *Faecalibacterium prausnitzii*

**Supplementary Material 2: Supplementary Fig. 2**. *Fp*-EVs protect TNBS-induced colitis in mice. (**A**) Flow diagram illustrating the *Fp*-EV treatment schedule in TNBS-induced mice (5–6 mice/group). (**B**) Changes in body weight among groups. (**C**) Survival rate of TNBS and Fp-EVs treated mice (5–6 mice/group). (**D**) Representative images of the colon. (**E**) Comparison of colon length among groups. (**F**) Representative H&E-stained colon sections. Magnification 200×. Scale bars represent 100 μm. (**G**) Histopathological scores among the different groups. Data are presented as the mean ± SD (n = 5–6). ** p < 0.01, ***p < 0.001 and ****p < 0.0001 vs. TNBS group. TNBS: 2,4,6-trinitrobenzene sulfonic acid; *Fp*: *Faecalibacterium prausnitzii*

**Supplementary Material 3: Supplementary Fig. 3**. LYHBHI medium itself have no inhibitory effect on DSS-induced colitis. (**A**) Flow chart of animal experimental scheme (5–6 mice/group). (**B**) Changes of body weight among groups. (**C**) Changes of disease activity of mice post 3% DSS administration. (**D**) Representative images of the colon. (**E**) The comparison of colon length among groups. (**F**) Representative H&E-stained colon sections. Magnification 200×. Scale bars represent 100 μm. (**G**) The histopathology scores among different groups. Data were presented as mean ± SD (n = 5–6). ** p < 0.01, ***p and ****p < 0.0001 vs. DSS group. DSS: dextran sulfate sodium; *Fp*: *Faecalibacterium prausnitzii*

**Supplementary Material 4: Supplementary Table 1**. Primer sequence

**Supplementary Material 5: Supplementary Table 2**. Inflammatory signaling pathways involved in the proteomic analysis of *Fp*-EVs

## Data Availability

The datasets used and/or analyzed during the current study are available from the corresponding author on reasonable request.

## Abbreviations

ANOVA: One-way analysis of variance
DAI: Disease activity index
DAPI: 4′,6-diamidino-2-phenylindole
DSS: Dextran sulfate sodium
ELISAs: Enzyme-linked immunosorbent assays
EVs: Extracellular vesicles
*F. prausnitzii*: Faecalibacterium prausnitzii
*Fp*-EVs: EVs derived from *F. prausnitzii*
FBS: Foetal bovine serum
GI: Gastrointestinal
GM-CSF: Granulocyte-macrophage colony stimulating factor
GO: Gene Ontology
H&E: Haematoxylin and eosin
HO-1: Heme oxygenase-1
IBD: Inflammatory bowel disease
IECs: Intestinal epithelial cells
IFN: Interferon
IL: Interleukin
KEGG: Kyoto Encyclopedia of Genes and Genomes
LC‒MS/MS: Chromatography-Mass Spectrometry/ Mass Spectrometry
LPLs: Lamina propria lymphocytes
MAPK: Mitogen activated protein kinase
MPO: Myeloperoxidase
NF-κB: Nuclear factor-κB
Nrf2: Nuclear factor erythroid 2-related factor
NTA: Nanoparticle tracking analysis
PBS: Phosphate-buffered saline
PVDF: Polyvinylidene difluoride
RIPA: Radioimmunoprecipitation assay
ROS: Reactive oxygen species
SCFAs: Short-chain fatty acids
SDS-PAGE: Sodium dodecyl sulfate–polyacrylamide gel electrophoresis–polyacrylamide gel electrophoresis
TEM: Transmission electron microscopy
TGF: Transforming growth factor
TNF: Tumor necrosis factor
TUNEL: Transferase-mediated deoxyuridine triphosphate-biotin nick end labeling
ZO: Zona occludens
