# Supplementary figures and images for: F. prausnitzii-derived extracellular vesicles attenuate experimental colitis by regulating intestinal homeostasis in mice
Source: Microb Cell Fact. 2023 Nov 15;22:235. doi: 10.1186/s12934-023-02243-7 (PMC10648384; doi:10.1186/s12934-023-02243-7)

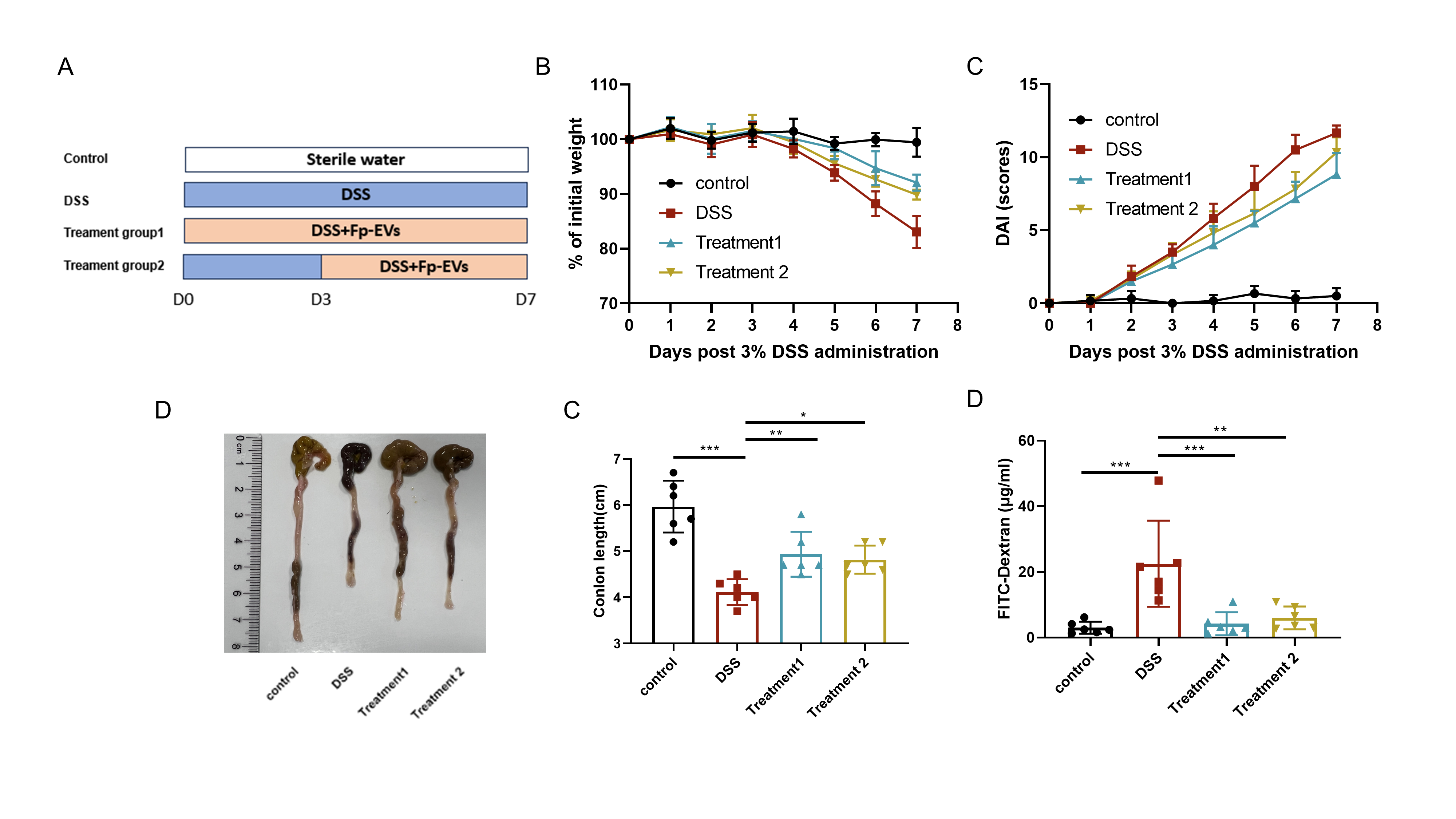

Supplement: Supplementary file 1 — Supplementary Material 1: Supplementary Fig. 1. Fp-EVs treat DSS-induced colitis in mice. (A) Flow diagram illustrating the Fp-EV treatment schedule in DSS-induced mice (5–6 mice/group). (B) Changes in body weight among groups. (C) Changes in the disease activity index of the mice after the administration of 3% DSS. (D) Representative images of the colon. (E) Comparison of colon length among groups. (F) The level of FITC-Dextran among groups. Data are presented as the mean ± SD (n = 5–6). ** p < 0.01, ***p < 0.001 and ****p < 0.0001 vs. DSS group. DSS: dextran sulfate sodium; Fp: Faecalibacterium prausnitzii [file 12934_2023_2243_MOESM1_ESM.png]

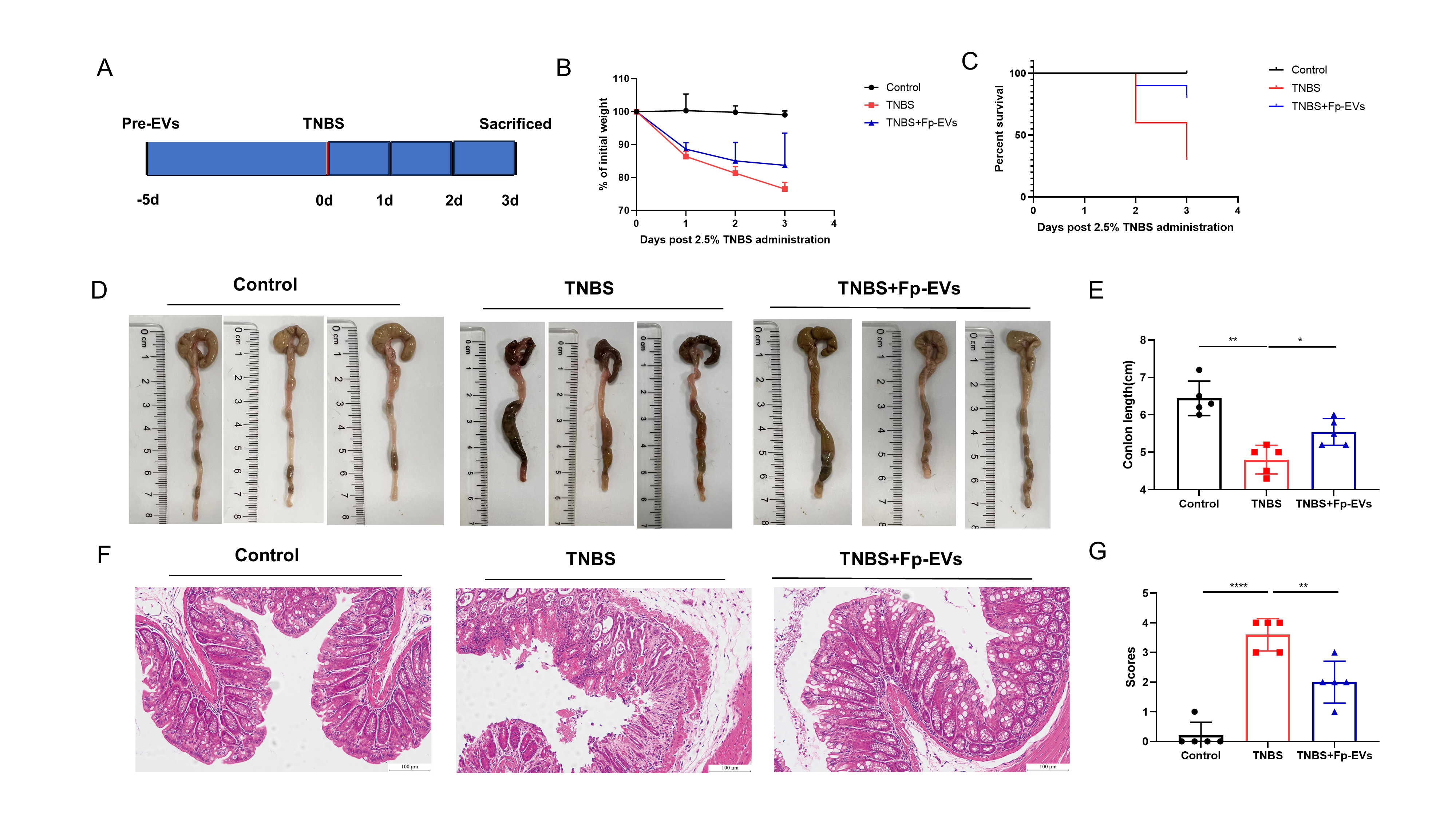

Supplement: Supplementary file 2 — Supplementary Material 2: Supplementary Fig. 2. Fp-EVs protect TNBS-induced colitis in mice. (A) Flow diagram illustrating the Fp-EV treatment schedule in TNBS-induced mice (5–6 mice/group). (B) Changes in body weight among groups. (C) Survival rate of TNBS and Fp-EVs treated mice (5–6 mice/group). (D) Representative images of the colon. (E) Comparison of colon length among groups. (F) Representative H&E-stained colon sections. Magnification 200×. Scale bars represent 100 μm. (G) Histopathological scores among the different groups. Data are presented as the mean ± SD (n = 5–6). ** p < 0.01, ***p < 0.001 and ****p < 0.0001 vs. TNBS group. TNBS: 2,4,6-trinitrobenzene sulfonic acid; Fp: Faecalibacterium prausnitzii [file 12934_2023_2243_MOESM2_ESM.png]

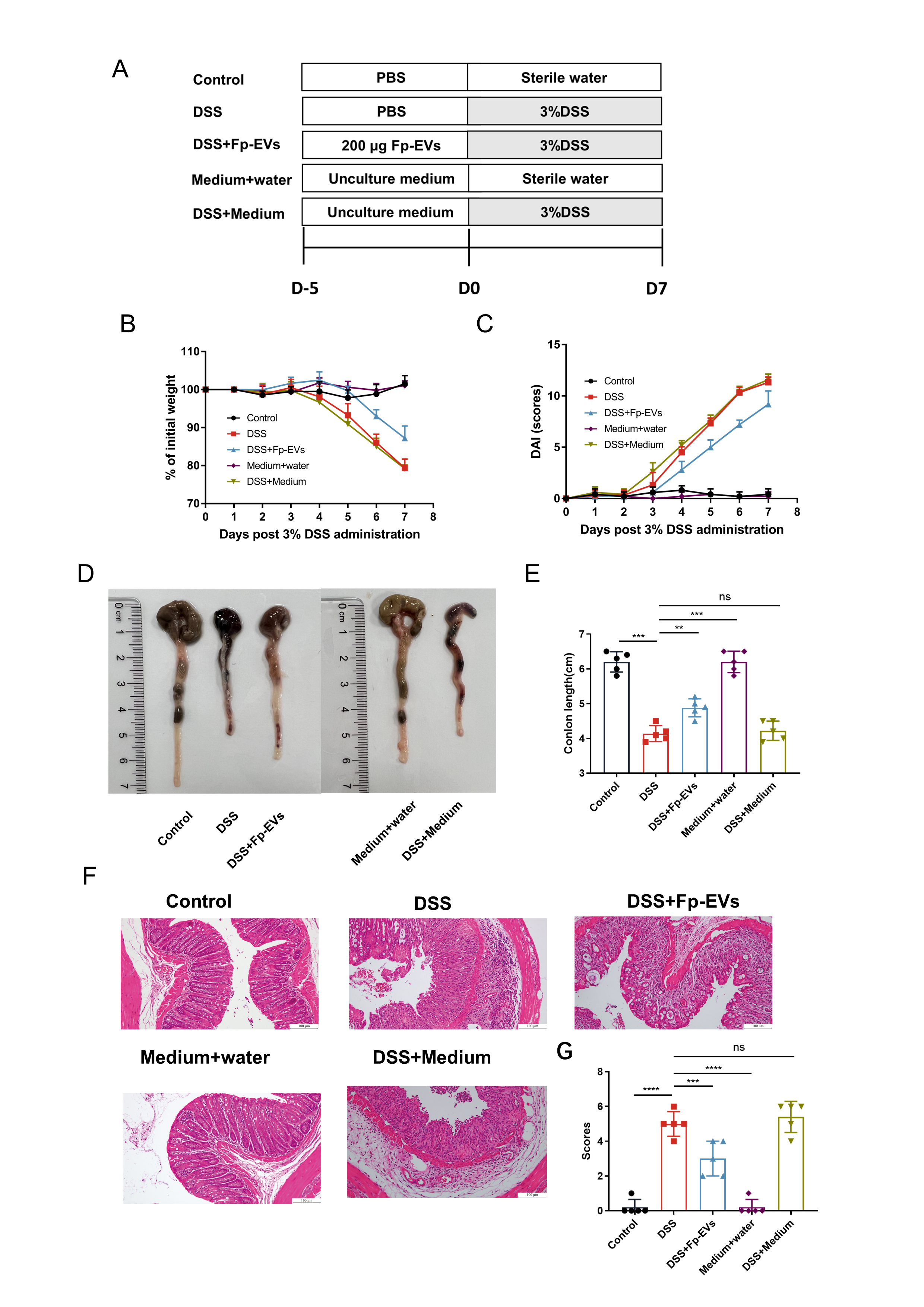

Supplement: Supplementary file 3 — Supplementary Material 3: Supplementary Fig. 3. LYHBHI medium itself have no inhibitory effect on DSS-induced colitis. (A) Flow chart of animal experimental scheme (5–6 mice/group). (B) Changes of body weight among groups. (C) Changes of disease activity of mice post 3% DSS administration. (D) Representative images of the colon. (E) The comparison of colon length among groups. (F) Representative H&E-stained colon sections. Magnification 200×. Scale bars represent 100 μm. (G) The histopathology scores among different groups. Data were presented as mean ± SD (n = 5–6). ** p < 0.01, ***p and ****p < 0.0001 vs. DSS group. DSS: dextran sulfate sodium; Fp: Faecalibacterium prausnitzii [file 12934_2023_2243_MOESM3_ESM.png]
