# Supplementary material for: F. prausnitzii-derived extracellular vesicles attenuate experimental colitis by regulating intestinal homeostasis in mice
Source: Microb Cell Fact. 2023 Nov 15;22:235. doi: 10.1186/s12934-023-02243-7 (PMC10648384; doi:10.1186/s12934-023-02243-7)
Supplement: Supplementary file 4 — Supplementary Material 4: Supplementary Table 1. Primer sequence [file 12934_2023_2243_MOESM4_ESM.docx]

Supplementary Table1. Primer sequence

| Gene name | Forward sequence | Reverse sequence |
| --- | --- | --- |
| β-actin | TATTGGCAACGAGCGGTTCC | GGCATAGAGGTCTTTACGGATGTC |
| IL-1β | AAGGGGACATTAGGCAGCAC | ATGAAAGACCTCAGTGCGGG |
| IL-2 | GAGCAGGATGGAGAATTACAGGAAC | TCCAGAACATGCCGCAGAGG |
| IL-4 | AGTTGTCATCCTGCTCTTCTTTCTC | ATGGCGTCCCTTCTCCTGTG |
| IL-6 | GAAATGATGGATGCTACCAAACTG | GACTCTGGCTTTGTCTTTCTTGTT |
| IL-10 | GCTCTTACTGACTGGCATGAG | CGCAGCTCTAGGAGCATGTG |
| IL-12a | TTTGATGATGACCCTGTGCCTTG | TTCTGAAGTGCTGCGTTGATGG |
| IL-17a | TTTAACTCCCTTGGCGCAAAA | CTTTCCCTCCGCATTGACAC |
| IFN-γ | GGAGGAACTGGCAAAAGGATGG | CAGGTGTGATTCAATGACGCTTATG |
| TNF | CCCTCACACTCACAAACCACC | CTTTGAGATCCATGCCGTTG |
| GM-CSF | TTGAACATGACAGCCAGCTACTAC | AATCCGCATAGGTGGTAACTTGTG |
| TGF-β | ACCGCAACAACGCCATCTATGAG | GGCACTGCTTCCCGAATGTCTG |
| HO-1 | ACCGCCTTCCTGCTCAACATTG | CTCTGACGAAGTGACGCCATCTG |
| Nrf2 | AAGCACAGCCAGCACATTCTCC | TGACCAGGACTCACGGGAACTTC |
