# Supplementary material for: F. prausnitzii-derived extracellular vesicles attenuate experimental colitis by regulating intestinal homeostasis in mice
Source: Microb Cell Fact. 2023 Nov 15;22:235. doi: 10.1186/s12934-023-02243-7 (PMC10648384; doi:10.1186/s12934-023-02243-7)
Supplement: Supplementary file 5 — Supplementary Material 5: Supplementary Table 2. Inflammatory signaling pathways involved in the proteomic analysis of Fp-EVs [file 12934_2023_2243_MOESM5_ESM.docx]

Supplementary Table2. Inflammatory signaling pathways involved in the proteomic analysis of *Fp*-EVs.

| **Level1** | **Level2** | **Map_ID** | **Map_Name** | **Test** |
| --- | --- | --- | --- | --- |
| Environmental Information Processing | Signal transduction | ko02020 | Two-component system | 15 |
| Environmental Information Processing | Signal transduction | ko04016 | MAPK signaling pathway - plant | 1 |
| Environmental Information Processing | Signal transduction | ko04066 | HIF-1 signaling pathway | 10 |
| Environmental Information Processing | Signal transduction | ko04151 | PI3K-Akt signaling pathway | 2 |
| Environmental Information Processing | Signal transduction | ko04152 | AMPK signaling pathway | 5 |
